# Efficacy, safety, and side effects of oliceridine in acute postoperative pain, a protocol for a systematic review and meta-analysis

**DOI:** 10.1371/journal.pone.0299320

**Published:** 2024-02-29

**Authors:** Anne Wolf, Matthias Unterberg, Andrea Witowski, Michael Adamzik, Alexander Wolf

**Affiliations:** 1 Institute of Anatomy and Clinical Morphology, University Witten/Herdecke, Witten, Germany; 2 Department of Anesthesiology, Intensive Care and Pain Medicine, University Hospital Knappschaftskrankenhaus Bochum and Ruhr University Bochum, Bochum, Germany; Sao Paulo State University (UNESP), Botucatu Medical School, BRAZIL

## Abstract

This will be the first meta-analysis on the efficacy, safety, and side effects of oliceridine on postoperative pain. Our aim with this work is to evaluate the clinical utility of this relatively new substance in a broad postoperative context. Oliceridine is a new so-called bias opioid that is approved for severe pain requiring an opioid. Due to its biased agonism, it is said to have fewer side effects than conventional opioids. This systematic review and meta-analysis will analyze the efficacy, safety, and side effects of oliceridine compared to placebo or morphine in acute postoperative pain for up to 72 hours. In January 2024, an extensive search in various databases will be performed without restrictions for randomized controlled trials with at least single blinding. After data extraction, data will be pooled and meta-analytic calculations performed. A random-effects model will be used. Dichotomous data will be presented as risk ratio and continuous data as standardized mean difference. Dose-dependent side effects will be evaluated with meta-regression. Heterogeneity will be assessed via the Q statistic and prediction interval in case of a sufficient number of included studies. Publication bias will be examined using funnel plot and Duval and Tweedie’s trim and fill method.

## Introduction

Oliceridine is a newly developed opioid that has already been approved in 2020 in the United States by the Food and Drug Administration (FDA) for the intravenous treatment of acute opioid-requiring pain [1]. Oliceridine has a fast onset time. Analgesia sets in after only 5 to 10 minutes and a maximum plasma level is reached after one administration after 10 minutes. After a one-hour infusion, oliceridine has a half-life of 1.6 to 2.7 hours. The drug is metabolized via the liver isoenzymes CYP2D6 and CYP3A4 [2]. Neither mild nor moderate liver insufficiency nor renal insufficiency necessitates dose adjustment [3]. These properties make oliceridine a suitable analgesic for severe pain in almost all patients.

The special feature of this new opioid is the biased agonism [4]. Conventional opioids, such as morphine, bind to the μ-opioid-receptor and activate the G-protein and the ß-arrestin pathway. The G-protein cascade leads to pain relief, while activation of the ß-arrestin cascade causes negative side effects such as, e.g., nausea, vomiting, or constipation. The unique property of oliceridine is to activate the G-protein cascade while activating the ß-arrestin pathway to a much lesser extent and so leading to fewer side effects.

In postoperative pain management, the practitioner is often faced with moderate to severe acute pain [5]. In postoperative pain therapy, opioids are used in combination with nonopioid analgesics and co-analgesics [6]. This usually leads to pain relief. However, ’conventional’ opioids sometimes have a pronounced side effect profile, as mentioned above, which may require further therapy [7]. For example, nausea and vomiting are treated with antiemetics and constipation with peripheral opioid receptor antagonists [8]. Respiratory depression may result in respiratory support, admission to the ICU, or antagonization with return of pain [9].

All these countermeasures increase costs and can prolong the length of stay in the post-anesthesia care unit (PACU) or even the hospital length of stay [10–12]. In high-risk patients there is a savings potential of 363,944 $ per 1,000 patients with oliceridine [13]. Therefore, avoiding these negative effects of pain therapy while administering sufficient analgesia is highly favorable for patients, caregivers, hospitals and health insurance providers.

The purpose of the planned systematic review and meta-analysis is to investigate the efficacy of a novel biased opioid oliceridine compared to placebo and morphine as a reference substance in moderate to severe postoperative pain. In addition, we will examine the occurrence of side effects and safety compared to placebo and morphine. To date there has been no systematic review or meta-analysis investigating the efficacy, safety, and side effects of oliceridine.

## Materials and methods

The meta-analysis will be performed and reported in accordance with the current Preferred Reporting Items for Systematic reviews and Meta-Analyses (PRISMA-P) statement and protocol [14]. The PRISMA-P-checklist can be found as S1 Checklist. The study was conceptualized by AnWo, MiAd, and AlWo. The protocol was registered on the International Platform of Registered Systematic Reviews and Meta-analysis Protocols (INPLASY; http://inplasy.com) with the registration number INPLASY202310063.

### Eligibility criteria

Using the PICO framework [15] we can describe the study as follows.

Patients: Postoperative patients (up to 72 hours) with moderate to severe pain

Intervention: Oliceridine (TRV130)

Comparison: Morphine and Placebo

Outcome: Efficacy (pain reduction), safety, or side effects

### Information sources

The databases Pubmed/Medline, Scopus, Cochrane Central Register of Controlled Trials (CENTRAL), Web of Science, and Google Scholar as sources for gray literature will be used. CENTRAL also includes the databases Excerpta Medica Database (Embase), the Cumulative Index to Nursing and Allied Health Literature (CINAHL), ClinicalTrials.gov, and the World Health Organization’s International Clinical Trials Registry Platform (ICTRP).

### Search strategy

An extensive literature search in English will be performed in January 2024 by two independent researchers (AlWo, AnWo) using the Medical Subject Heading (MeSH) “(3-methoxythiophen-2-yl) methyl) ((2-(9- (pyridin-2-yl) -6-oxaspiro (4.5) decan-9-yl) ethyl))amine” in Pubmed and MeSH entry terms “oliceridine”, “olinvyk”, “TRV130” and “TRV-130” (please see S1 File for further details) without restrictions in all databases to identify all relevant literature. Additionally, all reviews and clinical trials will be screened for additional relevant literature.

### Study records and data items

The identified literature will be stored in the Endnote 20 reference management program (Clarivate Analytics, Philadelphia, USA). Duplicates will be removed, and so studies with duplicates will only be considered once. Two researchers (AlWo, AnWo) will independently identify eligible randomized controlled trials with at least single blinding reporting any data listed in Table 1 of the collected references. The results of each researcher will be compared and, in case of discrepancies, a third party (MaUn) will be involved for decision making.

**Table 1:** Data that will be extracted from the included trials.

Basic study characteristics	Patient and treatment characteristics	Outcome parameters
Study registry identifier First author Publication year Year of data collection Country of study Funding source Study phase Inclusion/Exclusion criteria	Age Sex Type of anesthesia Pain therapy concept Starting dose Duration of treatment	Analgesia efficacy reduction in pain perception response to study medication cumulative opioid consumption Respiratory safety events Hypoxia Hypoventilation Respiratory depression Adverse events & serious adverse events Mortality Headache Dizziness Somnolence Sedation Anxiety Nausea Vomiting Constipation Pruritus General pruritus Hyperhidrosis Hot flush

Data from included trials will be extracted and transferred to an MS Excel sheet (Microsoft, Redmond, Washington, USA) by two researchers independently (AlWo and MaUn) and compared. In case of disagreement, a third party will be involved (AnWi). For data that will be extracted from the trial publications, please see Table 1.

### Outcomes and prioritization

The primary outcome of this systematic review and meta-analysis is the efficacy of olicerdine compared to morphine and placebo. Morphine is the reference substance in pain therapy. To demonstrate efficacy, Oliceridine must not be inferior to the reference substance. A comparison against a placebo also makes sense, as the placebo group can also access a pain rescue analgesic. Any form of analgesic efficacy reported in the studies will be recorded. Analgesic efficacy will be regularly measured in terms of change in the numeric rating scale as a continuous variable (NRS, with values ranging from 0 = no pain to 10 = greatest pain imaginable), or as a dichotomous variable reflecting treatment success according to predefined criteria.

As a biased opioid, oliceridine should have an improved safety and side-effect profile. For that reason, we will analyze as secondary outcomes respiratory safety events, hypoxia, hypoventilation, or respiratory depression, as this is a relevant concern of opioid treatment compared to controls. Further, we will analyse cumulative reported adverse and serious adverse events as well as mortality. The occurrence of other typical side effects of opioids such as nausea, vomiting, constipation, dizziness, headache, somnolence, sedation, anxiety, pruritus, general pruritus, hyperhidrosis, or hot flush will also be compared.

### Risk of bias in individual studies

The risk of bias will be evaluated with the Cochrane Risk of Bias 2 (RoB 2) tool [16], which covers the domains random sequence generation, allocation concealment, blinding, attrition bias, reporting bias, and other bias by two researchers (AnWi, MaUn) independently.

### Data synthesis

For data synthesis, we will use the Comprehensive Meta-Analysis Version 4.0 Professional software (Biostat Inc, Englewood, NJ; USA).

All calculations will be executed with the random-effects model. Dichotomous data will be calculated as the risk ratio (RR) with a 95% confidence interval, continuous data will be calculated as standardized mean differences (SMD) based on Hedges’s g and its 95% confidence interval. Hedges’s g is an adjustment of Cohen’s d. Cohen’s d tends to overestimate the absolute value of δ in small samples, which can be resolved using Hedges’s g.

The consistency of the data will be validated using the I^2^ statistic. Heterogeneity will be assessed by calculation of the Q statistic. Furthermore, we will calculate the prediction interval as a measure of heterogeneity if there are a sufficient number of studies to be included in this analysis [17].

In case of studies of different methodological quality (e.g. single vs. double-blind), a sensitivity analysis will be performed to verify the impact of this study(s) on the overall outcome. We investigate the dose-dependent effects on efficacy and side effects of oliceridine. We will do this using both subgroup analyses and meta-regression. We consider this important because of the postulated reduced side effects, whether side effects increase with increasing dose or not. For meta-regression, we will use method of the moment with Knapp-Hartung adjustment as a conservative approach. R^2^ will be calculated as measure of correlation. All formulas can be found here [18, 19].

### Meta-bias

Publication bias will be examined using the funnel plot and the Duval and Tweedie’s trim and fill method [20].

### Confidence in cumulative evidence

Result interpretation and recommendations will be based on the Grading of Recommendations, Assessment, Development and Evaluations (GRADE) framework [21].

Potential amendments to the study protocol will lead to an immediate update in the meta-analysis registry, as well as information to the journal regarding changes for an update on the published protocol. After data collection and meta-analytic calculations, we plan to publish the results in an international peer-reviewed journal.

## Discussion

Oliceridine seems to be a promising analgesic with an improved side-effect profile compared to conventional non-biased opioids. To further evaluate the efficacy, safety, and side effects of this drug, we will perform this meta-analysis.

To ensure the most holistic search possible, we will search several databases. Especially CENTRAL also covers various databases such as Pubmed, Embase, CINAHL, ClinicalTrials.gov and the World Health Organization International Clinical Trials Registry Platform (ICTRP). The search terms are also broad, with only the drug name and synonyms. Oliceridine has been approved by the FDA since 2020. With this open-search strategy, we expect no more than an average of 250 hits per database and can thus detect almost all published studies with a high probability.

We will include patients in a time frame of up to 72 hours postoperatively. Pain perception is greatest in the first three days after surgery. This is also associated with analgesic or opioid use. Subsequently, both pain and opioid consumption decrease [22, 23]. By examining this time window, we are more likely to capture postoperative acute pain and thus understand the efficacy, safety, and side effects of oliceridine.

In addition to study and population-specific data from patients, we will extract a multitude of relevant opioid-related side effects. Opioid-related side effects may limit its use and may cause a negative patient experience. There are indications that there is an increasing dose-dependent occurrence of side-effects [2]. To address this issue, we will perform a subgroup analysis and a meta-regression regarding the initial dose and the time-dependent cumulative dose.

We will use Comprehensive Meta-Analysis (CMA) for data synthesis in the current version. CMA has the advantage over other meta-analysis software to allow data input in various formats which are then automatically transformed into the effect size. Another advantage is the automatically generated prediction interval. Apart from the mean effect size with its confidence interval from current studies, the prediction interval tells us the true effect size of any population and should be presented in each meta-analysis [17].

Many meta-analyses use the fixed or random effect model based on statistical heterogeneity based on the I^2^ statistics. This approach is inappropriate [24]. We assume a natural heterogeneity based on varying drug dosing, surgery, anesthesia, and populations. Thus, we will use a random-effects model to generate generalizable results.

As effect size for dichotomous data, we will use the risk ratio (RR). Compared to the odds ratio (OR), the RR is more clinically intuitive [25] and seems to us and the potential readership appropriate. As effect size for continuous or mixed data we will use Hedges’s g, a slightly modified version of Cohen D that provides a better estimate as the difference in estimates converges with larger numbers [26].

This will be the first meta-analysis to evaluate the efficacy and effectiveness of oliceridine compared to placebo or morphine in postoperative acute pain. Our aim with this work is to evaluate and classify the clinical utility of this relatively new substance in a broad postoperative context.

## Supporting information

S1 Checklist PRISMA-P (Preferred Reporting Items for Systematic review and Meta-Analysis Protocols) 2015 checklist: Recommended items to address in a systematic review protocol*. (DOC)

S1 File Search terms and strategy. (DOCX)
