# Supplementary material for: Efficacy, safety, and side effects of oliceridine in acute postoperative pain, a protocol for a systematic review and meta-analysis
Source: PLoS One. 2024 Feb 29;19(2):e0299320. doi: 10.1371/journal.pone.0299320 (PMC10903901; doi:10.1371/journal.pone.0299320)
Supplement: S1 Checklist — (DOC) [file pone.0299320.s001.doc]

**PRISMA-P (Preferred Reporting Items for Systematic review and Meta-Analysis Protocols) 2015 checklist: recommended items to address in a systematic review protocol***

| Section and topic | Item No | Checklist item |
| --- | --- | --- |
| ADMINISTRATIVE INFORMATION | | |
| Title: |  |  |
| Identification | 1a | Efficacy, safety and side-effects of Oliceridine in acute postoperative pain, a protocol for a systematic review and meta-analysis |
| Update | 1b | N/A |
| Registration | 2 | International Platform of Registered Systematic Reviews and Meta-analysis Protocols INPLASY202310063 |
| Authors: |  |  |
| Contact | 3a | Dr. Anne Wolf, Institute of Anatomy and Clinical Morphology, University Witten/Herdecke, Witten, Germany; [anne.wolf@uwh.de](mailto:Anne.wolf@uwh.de)  Dr. Matthias Unterberg, Department of Anesthesiology, Intensive Care and Pain Medicine, University Hospital Knappschaftskrankenhaus Bochum and Ruhr University Bochum, Bochum, Germany; [matthias.unterberg@kk-bochum.de](mailto:matthias.unterberg@kk-bochum.de)  Dr. Andrea Witowski, Department of Anesthesiology, Intensive Care and Pain Medicine, University Hospital Knappschaftskrankenhaus Bochum and Ruhr University Bochum, Bochum, Germany; [andrea.witowski@kk-bochum.de](mailto:andrea.witowski@kk-bochum.de)  Prof. Dr. Michael Adamzik), Department of Anesthesiology, Intensive Care and Pain Medicine, University Hospital Knappschaftskrankenhaus Bochum and Ruhr University Bochum, Bochum, Germany; [michael.adamzik@kk-bochum.de](mailto:michael.adamzik@kk-bochum.de)  Dr. Alexander Wolf, Department of Anesthesiology, Intensive Care and Pain Medicine, University Hospital Knappschaftskrankenhaus Bochum and Ruhr University Bochum, In der Schornau 23-25, 44869 Bochum, Germany; [alexander.wolf-x5i@ruhr-uni-bochum.de](mailto:alexander.wolf-x5i@ruhr-uni-bochum.de) |
| Contributions | 3b | Dr. Anne Wolf: conceptualization, study search and identification, writing - original draft preparation  Dr. Matthias Unterberg: data extraction, risk of bias assessment, writing - original draft preparation  Dr. Andrea Witowski: risk of bias assessment, writing—review and editing  Prof. Michael Adamzik: conceptualization, writing—review and editing  Dr. Alexander Wolf: conceptualization, study search and identification, data extraction and synthesis, writing - original draft preparation, review and editing, guarantor |
| Amendments | 4 | N/A |
| Support: |  |  |
| Sources | 5a | Departmental resources only |
| Sponsor | 5b | N/A |
| Role of sponsor or funder | 5c | N/A |
| INTRODUCTION | | |
| Rationale | 6 | Oliceridine is a novel so called bias opioid which is approved for severe pain requiring an opioid. In recent trials there seems to be a sufficient efficacy in pain reduction with an optimized side-effect profile. |
| Objectives | 7 | Patients: Postoperative (up to 72hours) patients with moderate to severe pain  Intervention: Oliceridine (TRV130)  Comparison: Morphine and Placebo  Outcome: Efficacy (pain reduction), safety, or side effects |
| METHODS | | |
| Eligibility criteria | 8 | Randomized controlled trials with at least single blinding examining oliceridine for postoperative pain and using morphine and/or placebo as comparison, without further restrictions regarding publication year or language |
| Information sources | 9 | The databases Pubmed/Medline, Scopus, Cochrane Central Register of Controlled Trials (CENTRAL; including Embase, CINAHL, ClinicalTrials.gov, ICTRP), Web of Science, and Google Scholar as source for gray literature will be used |
| Search strategy | 10 | Search terms: “oliceridine”, “TRV-130”, “TRV130” without any restrictions |
| Study records: |  |  |
| Data management | 11a | The identified literature will be stored in the reference management program Endnote 20 and duplicates removed. |
| Selection process | 11b | Two researchers will independently identify eligible studies reporting relevant data from the collected references. The results of each researcher will be compared and in case of discrepancies, a third party will be involved for decision making. |
| Data collection process | 11c | Data from included trials will be extracted and transferred to an MS Excel sheet (Microsoft, Redmond, Washington, USA) by two researchers independently and compared. In case of disagreement a third party will be involved. |
| Data items | 12 | Study registry identifier; First author; Publication year; Year of data collection; Country of study; Funding source; Study phase; Inclusion/Exclusion criteria; Type of Anesthesia; Pain therapy concept; Age; Sex; Starting dose; Cumulative opioid consumption; Duration of treatment; Analgesia efficacy (reduction in pain perception, response to study medication); Respiratory safety events; Hypoxia; Hypoventilation; Respiratory depression; Adverse events; Serious adverse events; Mortality; Nausea; Vomiting; Constipation; Dizziness; Headache; Somnolence; Sedation; Anxiety; Pruritus; General pruritus; Hyperhidrosis; Hot flush |
| Outcomes and prioritization | 13 | Primary outcome: Analgesia efficacy (reduction in pain perception, response to study medication)  Secondary outcome: Safety and side-effects (Respiratory safety events; Hypoxia; Hypoventilation; Respiratory depression; Adverse events; Serious adverse events; Mortality; Nausea; Vomiting; Constipation; Dizziness; Headache; Somnolence; Sedation; Anxiety; Pruritus; General pruritus; Hyperhidrosis; Hot flush)  Subgrouping/meta-regression of efficacy, safety and side-effects in context of starting opioid dose and cumulative opioid dose |
| Risk of bias in individual studies | 14 | The risk of bias will be assessed with the Cochrane Risk of Bias 2 (RoB 2) tool, which covers the domains random sequence generation, allocation concealment, blinding, attrition bias, reporting bias and other bias by two researcher independently. In case of study of different methodological quality (e.g., single vs. double-blind), a sensitivity analysis will be performed to verify the impact of this study(s) on the overall outcome. |
| Data synthesis | 15a | All calculations will be executed with the random effects model. Dichotomous data will be calculated as risk ratio (RR) with a 95% confidence interval, continuous data data will be calculated as standardized mean differences (SMD) based on Hedges’s g and its 95% confidence interval. |
|  | 15b | Consistency of the data will be validated using the I2 statistic. Heterogeneity will be assessed by calculation of the Q-statistic. Furthermore, we will calculate the prediction interval as a measure of heterogeneity if there is a sufficient number of studies to be included in this analysis |
|  | 15c | Subgrouping/meta-regression of efficacy, safety and side-effects in context of starting opioid dose and cumulative opioid dose; a sensitivity analysis will be performed in case of varying study quality with removing the lower quality studies assessing the effect on the overall outcome. |
|  | 15d | If a quantitative analysis is inappropriate, a descriptive presentation of the data will be chosen. |
| Meta-bias(es) | 16 | Publication bias will be examined using funnel plot and the Duval’s and Tweedie’s trim and fill method |
| Confidence in cumulative evidence | 17 | Result interpretation and recommendations will be based on the Grading of Recommendations, Assessment, Development and Evaluations (GRADE) framework |

*** It is strongly recommended that this checklist be read in conjunction with the PRISMA-P Explanation and Elaboration (cite when available) for important clarification on the items. Amendments to a review protocol should be tracked and dated. The copyright for PRISMA-P (including checklist) is held by the PRISMA-P Group and is distributed under a Creative Commons Attribution Licence 4.0.**

*From: Shamseer L, Moher D, Clarke M, Ghersi D, Liberati A, Petticrew M, Shekelle P, Stewart L, PRISMA-P Group. Preferred reporting items for systematic review and meta-analysis protocols (PRISMA-P) 2015: elaboration and explanation. BMJ. 2015 Jan 2;349(jan02 1):g7647.*
