# Supplementary material for: Efficacy, safety, and side effects of oliceridine in acute postoperative pain, a protocol for a systematic review and meta-analysis
Source: PLoS One. 2024 Feb 29;19(2):e0299320. doi: 10.1371/journal.pone.0299320 (PMC10903901; doi:10.1371/journal.pone.0299320)
Supplement: S1 File — (DOCX) [file pone.0299320.s002.docx]

Search terms and strategy

| Medical Subject Heading (MeSH) Term  Terms | "((3-methoxythiophen-2-yl)methyl)((2-(9-(pyridin-2-yl)-6-oxaspiro(4.5)decan-9-yl)ethyl))amine”  „OLICERIDINE“ OR  „TRV130“ OR  „TRV-130“ OR  “OLINVYK” |
| --- | --- |
| Strategy | |
| **MEDLINE/Pubmed**  <https://pubmed.ncbi.nlm.nih.gov/?otool=iderublib> | (((3-methoxythiophen-2-yl)methyl)((2-(9-(pyridin-2-yl)-6-oxaspiro(4.5)decan-9-yl)ethyl))amine[MeSH Terms]) OR (Oliceridine OR Olinvyk OR TRV130 OR TRV-130) |
| **Scopus**  <https://www.scopus.com/search/form.uri?display=basic> | ( CASREGNUMBER ( 1401028-24-7 ) OR TITLE-ABS-KEY ( oliceridine ) OR TITLE-ABS-KEY ( olinvyk ) OR TITLE-ABS-KEY ( trv130 ) OR TITLE-ABS-KEY ( trv-130 ) ) |
| **Cochrane (including PubMed, Embase, CT.gov, ICTRP)**  <https://www.cochranelibrary.com/advanced-search?cookiesEnabled> | (Oliceridine):ti,ab,kw OR (TRV130):ti,ab,kw OR (TRV-130):ti,ab,kw OR (olinvyk):ti,ab,kw |
| **Web of science**  <http://apps.webofknowledge.com/WOS_GeneralSearch_input.do?product=WOS&search_mode=GeneralSearch&SID=D6ozKVscVFF7Siwkh3R&preferencesSaved=> | ((ALL=(Oliceridine)) OR ALL=(Olinvyk)) OR ALL=(TRV130) OR ALL=(TRV-130) |
| **Google Scholar**  https://scholar.google.de | „OLICERIDINE“ OR „TRV130“ OR „TRV-130“ OR “OLINVYK” |

**Identification of studies via other methods**

**Identification of studies via databases and registers**

Records identified from:

Websites (n = )

Citation searching (n = )

etc.

Records removed *before screening*:

Duplicate records removed (n = )

Records removed for other reasons (n = )

Records identified from:

Pubmed/Medline (n = )

SCOPUS (n= )

CENTRAL (n = )

Web of Science (n = )

Google Scholar (n = )

**Identification**

Records screened

(n = )

Records excluded**

(n = )

Reports not retrieved

(n = )

Reports sought for retrieval

(n = )

Reports sought for retrieval

(n = )

Reports not retrieved

(n = )

**Screening**

Reports assessed for eligibility

(n = )

Reports excluded:

Reason 1 (n = )

Reason 2 (n = )

Reason 3 (n = )

etc.

Reports assessed for eligibility

(n = )

Reports excluded:

Reason 1 (n = )

Reason 2 (n = )

Reason 3 (n = )

etc.

Studies included in review

(n = )

Reports of included studies

(n = )

**Included**

*Adapted from:*  Page MJ, McKenzie JE, Bossuyt PM, Boutron I, Hoffmann TC, Mulrow CD, et al. The PRISMA 2020 statement: an updated guideline for reporting systematic reviews. BMJ 2021;372:n71. doi: 10.1136/bmj.n71. For more information, visit: <http://www.prisma-statement.org/>
